# Estimates and multivariable risk assessment of mid-buccal gingival recessions in an Italian adult population according to the 2018 World Workshop Classification System

**DOI:** 10.1007/s00784-022-04441-w

**Published:** 2022-03-18

**Authors:** Federica Romano, Stefano Perotto, Giacomo Baima, Gianfranco Macrì, Fabrizio Picollo, Mario Romandini, Giulia Maria Mariani, Mario Aimetti

**Affiliations:** 1 Periodontology Unit, C.I.R. Dental School, Department of Surgical Sciences, University of Turin, Via Nizza 230, 10126 Turin, Italy; 2 Postgraduate Program in Periodontology, C.I.R. Dental School, Department of Surgical Sciences, University of Turin, Turin, Italy; 3 Section of Graduate Periodontology, Faculty of Odontology, University Complutense, Madrid, Spain

**Keywords:** Epidemiology, Gingival recession, Periodontitis, Prevalence, Risk indicators

## Abstract

**Objectives:**

The aim of this cross-sectional study was to provide estimate of mid-buccal gingival recession (GR) according to the 2018 World Workshop Classification System and to explore GR risk indicators in a representative urban population in North-West of Italy.

**Material and methods:**

This is a secondary analysis using data collected in an epidemiological study enrolling a representative sample of 736 adults, living in Turin. GR prevalence was defined as the presence of at least one mid-buccal GR ≥ 1 mm. GRs were categorized according to the 2018 classification system (RT1, RT2, RT3) and to different severity cutoffs. Logistic regression analysis was performed to identify RT GR risk indicators.

**Results:**

Mid-buccal GR ≥ 1 mm affected 57.20% of subjects and 14.56% of teeth. When considering RT1 GRs, their prevalence was 40.90% and 6.29% at the patient and tooth level. RT2 and RT3 GRs affected 25.82% and 36.68% of the study population, respectively. RT1 GRs occurred mostly on maxillary and mandibular premolars and maxillary canines, while RT2 and RT3 GRs on maxillary molars and mandibular incisors. Older age, high education, and full-mouth plaque score (FMPS) < 30% were risk indicators for RT1 GRs, while older age, poor education, periodontitis, and FMPS > 60% were significant contributors to RT2 and RT3 GRs.

**Conclusions:**

RT1 and RT3 are fairly common findings in this Italian population and are significantly associated to different contributing factors and tooth type distribution pattern.

**Clinical relevance:**

Prevention strategies should target different socio-demographic, behavioral, and clinical risk indicators based on the RT classes.

**Supplementary Information:**

The online version contains supplementary material available at 10.1007/s00784-022-04441-w.

## Introduction

Gingival recession (GR) is defined as the exposure of the root surface due to the displacement of soft tissue margin beyond the cemento-enamel junction (CEJ) associated to clinical attachment loss [1]. GRs may be localized or generalized and can involve one or more surfaces. There are several potential factors that can lead to the apical shift of the gingival margin such as tooth malposition, thin scalloped phenotype, mechanical trauma, and plaque-induced inflammation [2]. Mid-buccal GR has received great attention in the scientific literature due to its negative impact on aesthetics and function but also due to the increased occurrence of carious and non-carious cervical and/or root lesions [3–5]. Indeed, periodontal plastic surgery procedures are applied to overcome these consequences by achieving complete root coverage [6–8].

The presence of GR at buccal sites has been reported as highly prevalent in a recent systematic review [9]. However, large variation exists across different countries. The prevalence of adult subjects presenting at least one GR ≥ 1 mm deep was 58% in the USA [10], 60.3% in Greece [11], 69.7% in Colombia [12], but higher in France (84.9%) [13] and Pomerania, a province in Eastern Germany (89.7%) [14], and included basically the whole sample in an urban area in Brazil (99.7%) [15]. For deeper GR in the 4–5-mm range, prevalence varied between 5.9 and 40.7% depending on the population surveyed [13, 15]. All these studies classified GR in terms of its vertical extension from the CEJ in millimeters using different severity thresholds.

Only one recent study [16] reported the prevalence of GR in the adult US population according to the 2018 World Workshop Classification System [1]. It distinguished among three GR types with reference to the amount of the interdental clinical attachment loss, as proposed by Cairo et al. in 2011 [17]:

- Recession type 1 (RT1) with no interproximal attachment loss;
- Recession type 2 (RT2) with the amount of attachment loss at interproximal sites being less or equal than that measured at the buccal site;
- Recession type 3 (RT3) with the amount of attachment loss at interproximal sites exceeding that at the buccal site.

Applying this classification to the NAHNES database, the estimated prevalence of RT1 recessions was 12.4%, which increased to 88.8% and 55.0% when considering RT2 and RT3 recessions, respectively [16].

Representative information about the occurrence and risk factors of GR according to the 2018 World Workshop Classification is lacking in Europe. Hence, the aims of this cross-sectional study were (1) to provide estimates of the prevalence of mid-buccal GR at the subject and tooth level considering both different severity cutoff values and the RT classification in a representative urban population in North-West Italy, and (2) to assess the association of potential risk indicators with the occurrence of GR in this population.

## Materials and methods

### Study design and population

This is a secondary analysis using existent data from a cross-sectional population-based epidemiological survey for periodontitis collected between December 1, 2009, and July 31, 2010, by the Section of Periodontology, C.I.R. Dental School, Department of Surgical Sciences, University of Turin (Italy) [18]. The Institutional Ethics Committee approved the study protocol (Approval No. 0082388), and subjects who agreed to participate signed an informed consent form in accordance with the Declaration of Helsinki.

All adults aged more than 18 years with main residency in Turin, one of the largest cities in North-West of Italy, were eligible for participation. The sample size for the original study was estimated assuming a 15% prevalence of severe periodontitis [19], a confidence level of 95%, and a precision of 2.5%. Considering a response rate of 50%, 1600 individuals were randomly selected and invited to participate. This representative sample of the target population was obtained by applying a stratified two-stage probability strategy to the Health Regional Register of Piedmont. In the first stage, primary sampling units were general practitioners randomly selected after stratification into the four health care districts of Turin. The second stage consisted on a random selection of subjects cared for by each practitioner. The sampling method and sample size calculation are described in details in a previous publication [18].

The flow chart of the study is summarized in Appendix Figure 1. The net random sample comprised 736 dentate individuals, aged between 20 and 75 years.

### Clinical examination

Patients enrolled into the study completed a self-administered questionnaire to collect information on socio-demographics, lifestyle factors (including educational level and smoking habit), and oral hygiene practices. Following completion, they were provided with a clinical examination that assessed their periodontal conditions. For consistency and to avoid inter-examiner variability, a single trained dental investigator performed all clinical examinations. Intra- and inter-examiner reliability for GR were assessed before the initiation of the study on 15 patients (387 teeth, 1161 buccal sites, 93 mid-buccal recession defects) at the medical office of a general practitioner not involved with the survey. Measurement of GR was performed twice with an interval of 24 h between the first and the second recording. Intra-examiner reliability revealed weighed (± 1 mm) *k* values of 0.96, and the inter-examiner agreement against a gold standard clinician was 0.87.

All fully erupted teeth, excluding third molars, were examined at six sites using a manual periodontal probe (PCPUNC15, Hu-Friedy®, Chicago, IL, USA). GR was defined as the distance from the CEJ to the free gingival margin (FGM), and probing depth (PD) represented the distance from FGM to the bottom of the sulcus/pocket. GR was scored as 0 if the FGM was located at the CEJ and was assigned a negative sign if the FGM was coronal to the CEJ. In sites where the CEJ was not detectable, the CEJ level was estimated on the basis of the adjacent teeth [20].

Clinical attachment level (CAL) was derived for each examined site by the sum of GR and PD. Measurements were rounded to the lower whole millimeter. Furthermore, the percentages of sites harboring plaque (full-mouth plaque score (FMPS)) or bleeding on probing (full-mouth bleeding score (FMBS)) were recorded. No radiographic examination was made.

Periodontal status was established using the case definitions developed jointly by the US Centres for Disease Control and Prevention (CDC) and the American Academy of Periodontology (AAP) to describe the prevalence of moderate and severe periodontitis in health surveys [21, 22]. The classification of no/mild periodontitis was assigned to cases that did not qualify as having moderate or severe periodontitis.

### Outcome definition

For the purpose of the study, only GRs at the mid-buccal sites were included in the analysis. Mid-buccal GRs were both categorized as ≥ 1 mm, ≥ 3 mm, ≥ 5 mm, and ≥ 7 mm according to their severity and as RT1, RT2, or RT3 using algorithms based on the operational definitions by Cairo et al. [17]. Furthermore, the following covariates were considered to define subpopulations and for the analytical epidemiological analysis:

- Subject level: age (categorized in three groups: 20—39 years, 40—59 years, 60—75 years), gender, educational level (categorized in three levels based on the Italian school system: low or primary and secondary school level, intermediate or high school diploma, high or education attainment beyond the high school level), periodontal status (no/mild periodontitis, moderate, severe periodontitis) [21, 22], smoking status (categorized in three levels: heavy smoker (≥ 10 cigarettes/day), light smoker (< 10 cigarettes/day), non-smoker) [23], FMPS (categorized in three levels : < 30%, 30 to 60%, > 60% using an approximation of the subject distribution into tertiles and considering a percentage of sites harboring plaque < 30% as a tolerable level of oral hygiene among the general population) [24, 25], FMBS (categorized in three levels: < 10%, 10 to 30%, > 30% according to the cutoff points for localized and generalized gingival inflammation) [26], self-reported toothbrushing frequency (categorized in 3 levels: not every day or once/day, twice/day, three or more times/day) [15], and professional scaling frequency (categorized in 2 levels: 12 months or less, more than 12 months) [16];
- Tooth level: tooth type (incisor, canine, premolar, molar), arch (maxilla, mandible), and mouth side (right, left).

### Statistical analysis

At subject level, the prevalence of GR was defined as the percentage of the population presenting at least one tooth with GR equal or higher than different depth thresholds (1, 3, 5, and 7 mm) or at least one mid-buccal site with RT1, RT2, or RT3 recession. Extent was classified as localized or generalized if GR ≥ 1 mm involved < 15% or ≥ 15% of teeth, respectively [16].

At tooth level, the prevalence of mid-buccal GR was calculated based on different vertical cutoff values and RT classes considering all teeth and then categorized by tooth type, arch, and side [27, 28]. Complex survey commands were used in all analyses to account for cluster correlations expected for the multistage sampling strategy used in the study.

A multiple multinomial logistic regression analysis was designed to assess the contribution of the independent variables to the probability of occurrence of RT1 or RT2/RT3 recessions in at least one tooth. Individuals without GR ≥ 1 mm were used as the reference group. Univariable models were fitted for each independent variable and those presenting *P* values < 0.25 were entered in the multivariable regression model. A backward selection procedure with a *P* value cutoff at 0.05 was used to identify the set of independent predictors. Using this approach, age, educational level, smoking habit, periodontal status, and FMPS remained in the final model. Due to the collinearity between FMPS and FMBS, only FMPS was maintained. Odds ratios (OR) were calculated with 95% CI. Data analysis was performed using SAS (version 9.4, SAS Institute) and SPSS (version 25, IBM) package.

## Results

### Prevalence and severity of mid-buccal gingival recessions

As summarized in Table 1, the presence of mid-buccal GR ≥ 1 mm was detected in more than half of the study population with 57.20% of subjects having at least one site affected and 33.56% having multiple recessions. Additionally, 42.12%, 8.83%, and 1.77% of the individuals presented at least one mid-buccal site with GR ≥ 3 mm, ≥ 5 mm, and ≥ 7 mm, respectively. The prevalence of GR increased with aging: in the age range 20–39, it was 34.18% and in the age group 60–75, it reached above 70%. It was also higher in heavy smokers, males, and individuals with severe periodontitis, mainly for higher thresholds of GR. Poor oral hygiene was associated to both GR severity and extent, whereas gingival inflammation only to severity.

**Table 1:** Prevalence of different gingival recession cut-off values for mid-buccal sites

	*n*	GR ≥ 1 mm (%)	*SE*	GR ≥ 3 mm (%)	*SE*	GR ≥ 5 mm (%)	*SE*	GR ≥ 7 mm (%)	*SE*	Generalized GR ≥ 1 mm (%)	*SE*
Total	736	57.20	1.82	42.12	1.82	8.83	1.05	1.77	0.49	33.56	1.74
Age groups (years)											
20–39	196	34.18	3.39	19.39	2.82	3.06	1.23	1.53	0.88	12.76	2.38
40–59	349	61.32	2.61	46.99	2.67	9.17	1.54	1.43	0.64	38.11	2.60
60–75	191	73.30	3.20	56.54	3.59	14.14	2.52	2.62	1.16	46.60	3.61
Gender											
Male	305	62.30	2.78	48.20	2.86	12.46	1.89	3.61	1.07	41.31	2.82
Female	431	53.60	2.40	37.82	2.34	6.26	1.17	0.46	0.33	28.07	2.16
Education level											
Low	305	61.64	2.78	49.18	2.86	14.43	2.01	2.30	0.86	38.69	2.79
Middle	284	55.99	2.95	38.38	2.89	5.99	1.41	2.11	0.85	30.99	2.74
High	147	50.34	4.12	34.69	3.93	2.72	1.34	0.00	0.00	27.89	3.70
Smoking status											
Non-smoker	488	56.97	2.24	42.42	2.24	8.20	1.24	0.82	0.41	32.99	2.13
Light smoker	116	53.45	4.63	33.62	4.39	4.31	1.89	0.86	0.86	27.59	4.15
Heavy smoker	132	61.36	4.24	48.48	4.35	15.15	3.12	6.06	2.08	40.91	4.28
Periodontal status											
No/mild periodontitis	168	28.57	3.49	14.29	2.70	0.60	0.59	0.00	0.00	10.12	2.33
Moderate periodontitis	289	54.33	2.93	34.26	2.79	3.81	1.13	0.35	0.35	25.61	2.57
Severe periodontitis	279	77.42	2.50	67.03	2.81	19.00	2.35	4.30	1.21	55.91	2.97
FMPS (%)											
< 30	122	47.54	4.52	25.41	3.94	4.10	1.79	0.82	0.82	22.13	3.76
30–60	277	58.58	2.96	41.16	2.96	5.42	1.36	0.36	0.36	32.49	2.81
> 60	337	59.64	2.67	48.96	2.72	13.35	1.85	3.26	0.97	38.58	2.65
FMBS (%)											
< 10	70	54.29	5.95	32.86	5.61	4.29	2.42	1.43	1.42	30.00	5.48
10–30	215	54.88	3.39	37.21	3.30	6.05	1.63	0.93	0.65	31.16	3.16
> 30	451	58.76	2.32	45.90	3.35	10.86	1.47	2.22	0.69	35.25	2.25
Toothbrushing frequency											
Once/day	107	56.07	4.80	47.66	4.83	11.21	3.05	0.93	0.92	36.45	4.65
Twice/day	315	57.14	2.79	40.00	2.76	7.94	1.52	2.54	0.89	30.79	2.60
At least 3 times/day	314	57.64	2.79	42.36	2.79	8.92	1.61	1.27	0.63	35.35	2.70
Scaling frequency											
≤ 12 months	325	61.23	2.70	45.54	2.76	7.08	1.42	1.23	0.61	36.92	2.68
> 12 months	411	54.01	2.46	39.42	2.41	10.22	1.49	2.19	0.72	30.90	2.28

*GR*, gingival recession; *SE*, standard error

The prevalence of GRs according to the types defined in the 2018 Classification System is reported in Table 2. RT1 and RT3 classes showed similar prevalence in the population (40.90% versus 36.68%), while RT2 was less frequently detected (25.82%). In particular, RT2 and RT3 recessions were more often found among individuals suffering from severe periodontitis, with low education and heavy smokers. Furthermore, they were more prevalent among those with higher percentage of plaque (FMPS > 30%) and generalized gingival inflammation (FMBS > 30%). When considering the distribution of interdental sulci/pockets in relation to PD severity and RT classes, PDs ≤ 3 mm were more frequently associated with RT2 than RT3 recessions (57% versus 39%), and moderate pockets of 4–5 mm were equally distributed (38% versus 37%), while severe pockets ≥ 6 mm deep were proportionately less prevalent at the interproximal sites of RT2 defects (5% versus 24%).

**Table 2:** Prevalence of mid-buccal gingival recession according to the RT classification system

	*N*	RT1 GRs (%)	*SE*	RT2 GRs (%)	*SE*	RT3 GRs (%)	*SE*
Total	736	40.90	1.81	25.82	1.61	36.68	1.78
Age groups (years)							
20–39	196	28.06	3.21	6.63	1.78	13.27	2.42
40–59	349	44.13	2.66	30.37	2.46	40.40	2.63
60–75	191	48.17	3.62	37.17	3.50	53.93	3.61
Gender							
Male	305	43.93	2.84	32.13	2.67	40.98	2.82
Female	431	38.75	2.35	21.35	1.97	33.64	2.28
Education level							
Low	305	37.70	2.78	34.10	2.71	45.57	2.85
Middle	284	43.31	2.94	22.18	2.47	34.51	2.82
High	147	42.86	4.08	15.65	3.00	22.45	3.44
Smoking status							
Non-smoker	488	41.39	2.23	24.80	1.95	36.27	2.18
Light smoker	116	41.38	4.57	18.97	3.64	29.31	4.23
Heavy smoker	132	38.64	4.24	35.61	4.17	44.70	4.33
Periodontal status							
No/mild periodontitis	168	27.98	3.46	1.79	1.02	2.38	1.18
Moderate periodontitis	289	41.52	2.90	16.96	2.21	30.10	2.70
Severe periodontitis	279	48.03	2.99	49.46	2.99	64.16	2.87
FMPS (%)							
< 30	122	41.80	4.47	13.11	3.06	17.21	3.42
30–60	277	46.21	3.00	22.38	2.50	34.30	2.85
> 60	337	36.20	2.62	32.23	2.57	45.70	2.71
FMBS (%)							
< 10	70	47.14	5.97	21.43	4.90	30.00	5.48
10–30	215	45.58	3.40	19.07	2.68	27.91	3.06
> 30	451	37.69	2.28	29.71	2.15	41.91	2.32
Toothbrushing frequency							
Once/day	107	31.78	4.50	35.51	4.63	42.06	4.77
Twice/day	315	39.05	2.75	25.71	2.46	35.56	2.70
At least 3 times/day	314	45.86	2.81	22.61	2.36	35.99	2.71
Scaling frequency							
≤ 12 months	325	47.69	2.78	25.23	2.41	37.54	2.69
> 12 months	411	35.52	2.36	26.28	2.17	36.01	2.37

*GR*, gingival recession; *RT*, recession type classification; *SE*, standard error

**Fig. 1:**
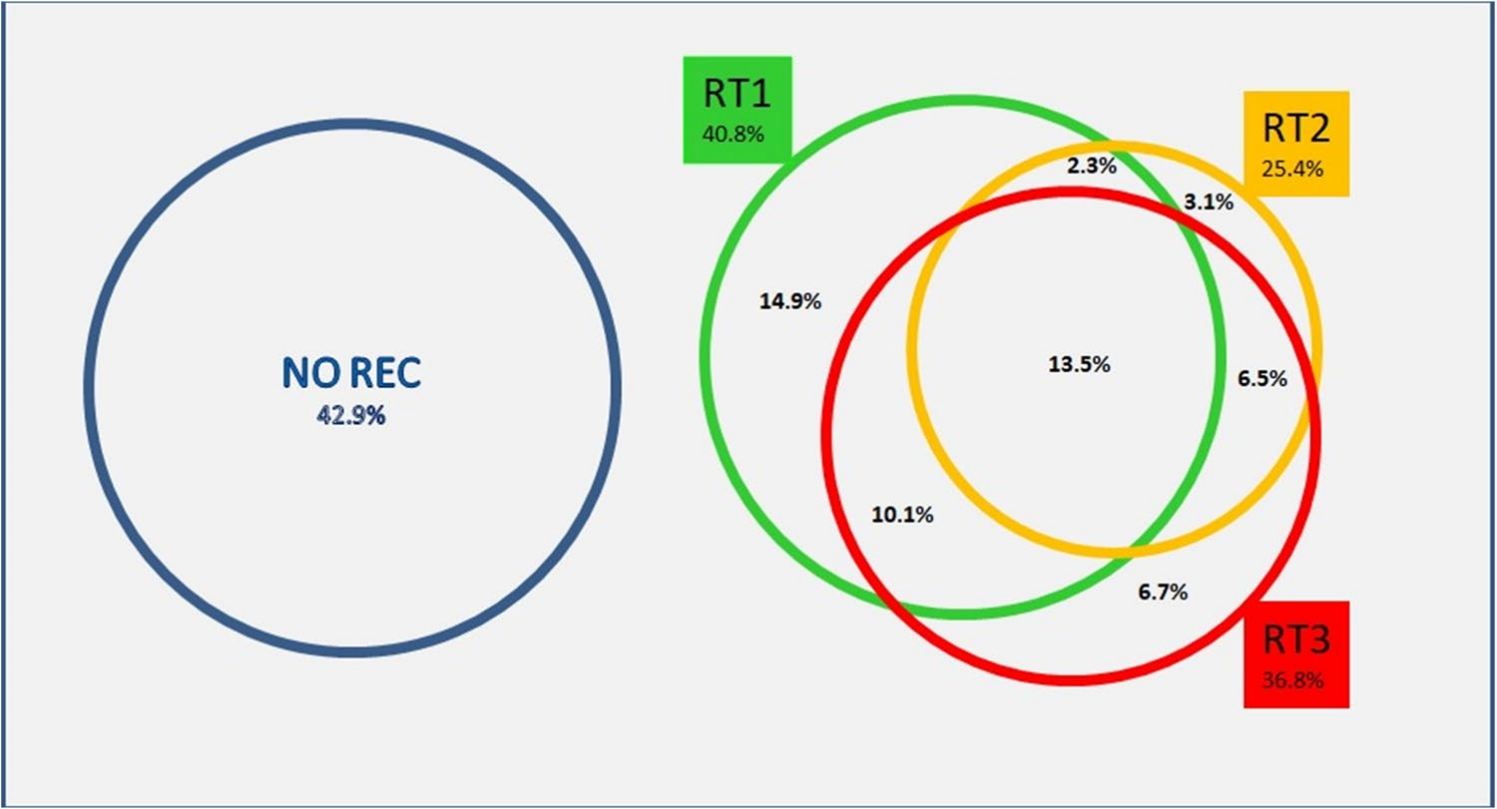
Distribution of RT classes of recession and their combination at the subject level

As depicted in Figure 1, only a minority of the subjects had exclusively one RT class of recession (14.9% RT1, 3.1% RT2, and 6.7% RT3), whereas the majority had a combination of them.

### Prevalence and severity of mid-buccal gingival recessions by tooth type

A total of 17,433 teeth were examined for the presence of GRs. Table 3 shows that 14.56% of them had the buccal surface affected by GR ≥ 1 mm and 6.79% by GR ≥ 3 mm, most of them being RT1 (prevalence of RT1: 6.29%). Recession thresholds ≥ 5 mm and ≥ 7 mm affected only a small percentage of the teeth. Higher frequency of GRs was found on the right side of the upper and lower jaw.

**Table 3:** Prevalence of mid-buccal gingival recessions according type of teeth

	*N*	GR ≥ 1 mm (%)	GR ≥ 3 mm (%)	GR ≥ 5 mm (%)	GR ≥ 7 mm (%)	RT1 GRs (%)	RT2 GRs (%)	RT3 GRs (%)
Total	17433	14.56	6.79	0.78	0.13	6.29	3.68	4.59
Tooth type								
Upper incisors	2684	10.54	3.50	0.11	0.00	4.88	2.79	2.87
Upper canines	1373	15.59	7.79	1.24	0.22	9.98	1.38	4.22
Upper premolars	2323	15.50	7.19	0.73	0.04	8.39	2.71	4.39
Upper molars	2219	16.04	8.52	1.31	0.23	3.97	6.53	5.64
Lower incisors	2784	17.49	8.33	1.61	0.43	5.75	5.21	6.54
Lower canines	1435	14.70	7.32	0.84	0.00	5.78	3.28	5.64
Lower premolars	2575	18.56	8.97	0.39	0.00	10.10	2.83	5.63
Lower molars	2040	7.35	2.89	0.15	0.05	2.11	3.63	1.62
Arch								
Maxilla	8599	14.11	6.48	0.77	0.10	6.41	3.51	4.19
Mandible	8834	15.01	7.10	0.79	0.15	6.18	3.84	4.99
Arch side								
Right	8731	15.81	7.40	0.84	0.11	6.78	3.93	5.10
Left	8702	13.32	6.18	0.72	0.14	5.80	3.42	4.09

*GR*, gingival recession

The distribution of GRs varied according to the type of teeth. Mandibular incisors, premolars, and maxillary molars presented the highest frequencies of GR ≥ 1 mm and ≥ 3 mm. Maxillary incisors and mandibular molars had the lowest prevalence of GR.

**Fig. 2:**
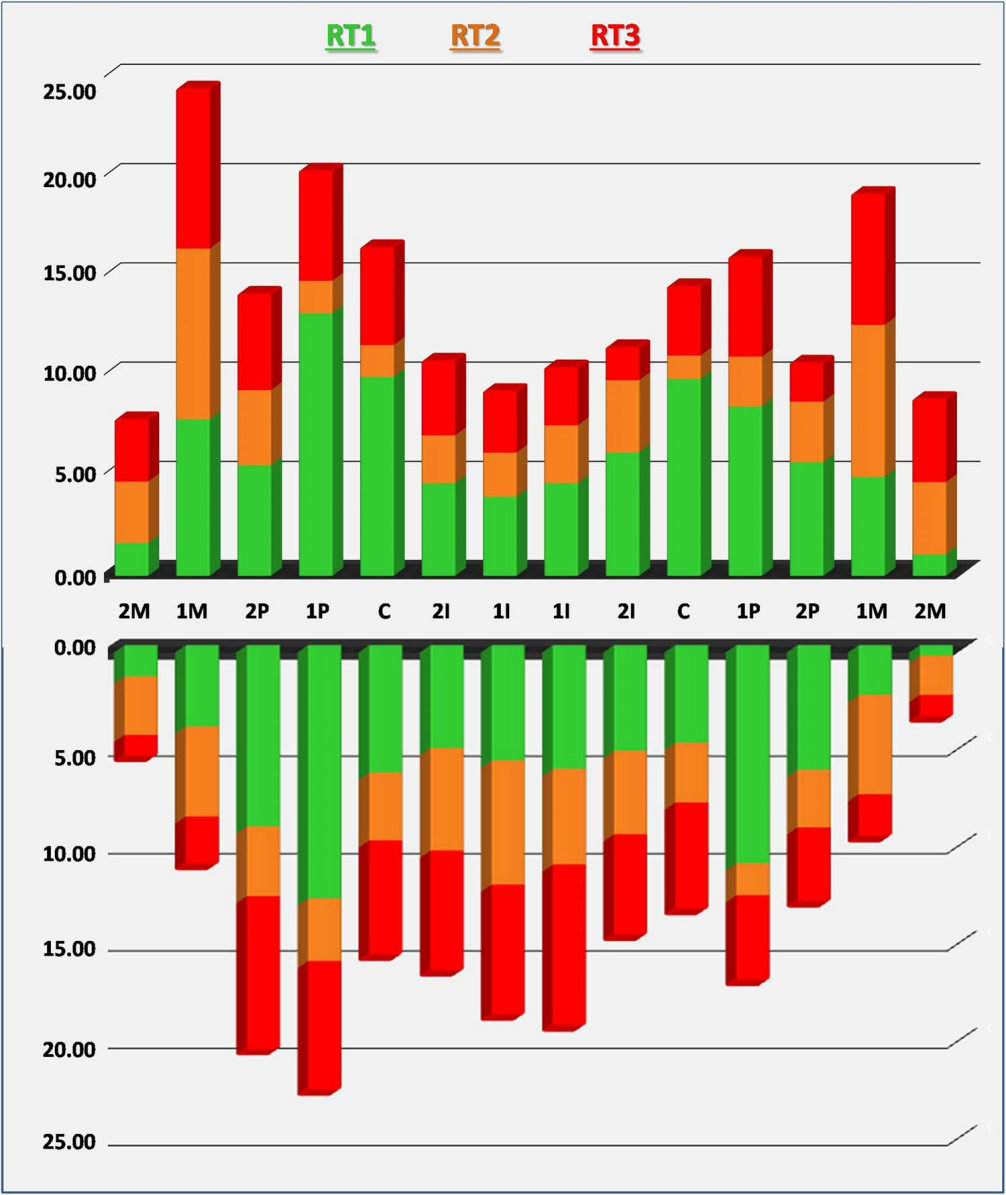
Prevalence of RT recessions according to tooth types

Among teeth with RT1, the most commonly affected were the mandibular premolars followed by the maxillary canines and premolars. Teeth showing the highest percentage of RT2 and RT3 recessions were the maxillary molars and the mandibular incisors. Figure 2 supports the greater involvement of the right side of the jaw with the maxillary first molar, the mandibular first and second premolars, and the maxillary first premolar being the most predisposed teeth.

### Factors associated with mid-buccal RT gingival recessions

In the multiple logistic regression analysis (Table 4), younger age and low educational level were significantly associated with lower chance of RT1 recessions at mid-buccal sites. Conversely, better performance in oral hygiene was significantly associated with the presence of RT1 recessions.

**Table 4:** Multiple multinomial regression models of factors associated with the presence of mid-buccal RT1 or mid-buccal RT2/RT3 gingival recessions in at least one tooth (adjusted for gender)

		OR	95% CI	*P* value
At least 1 RT1 recession (subjects without RT2 and RT3 recessions)				
	Age groups (years)			
	20–39	0.498	0.254–0.976	0.042
	40–59	0.752	0.405–1.394	0.365
	60–75	Ref	Ref	Ref
	Education level			
	Low	0.510	0.280–0.929	0.028
	Middle	0.819	0.479–1.399	0.465
	High	Ref	Ref	Ref
	Periodontal status			
	No periodontitis	1.022	0.490–2.133	0.954
	Moderate periodontitis	1.411	0.728–2.737	0.308
	Severe periodontitis	Ref	Ref	Ref
	FMPS (%)			
	< 30	2.401	1.229–4.692	0.010
	30–60	2.108	0.924–3.631	0.087
	> 60	Ref	Ref	Ref
At least 1 RT2 or RT3 recession				
	Age groups (years)			
	20–39	0.184	0.102–0.334	<0.001
	40–59	0.536	0.340–0.845	0.007
	60–75	Ref	Ref	Ref
	Education level			
	Low	1.866	1.087–3.204	0.024
	Middle	1.867	1.075–3.244	0.027
	High	Ref	Ref	Ref
	Periodontal status			
	No periodontitis	0.023	0.010–0.054	<0.001
	Moderate periodontitis	0.273	0.179–0.415	<0.001
	Severe periodontitis	Ref	Ref	Ref
	FMPS (%)			
	< 30	0.740	0.547–0.953	0.047
	30–60	1.238	0.812–1.886	0.322
	> 60	Ref	Ref	Ref

*GR*, gingival recession; *OR*, odds ratio; *95% IC*, 95% confidence interval; *ref*, reference

When considering RT2 and RT3 recessions, low and intermediate educational levels and suffering from severe periodontitis were significant risk indicators, while younger age and FMPS percentages < 30% had a protective effect. Self-reported frequency of toothbrushing, professional scaling frequency, and smoking habits were not significantly associated with the presence of GR.

## Discussion

This is the first epidemiological study focusing on mid-buccal GR in a representative sample of an adult Italian population according to both severity thresholds and RT classification system. GR ≥ 1 mm affected 57.20% of this population with 35% of them suffering from multiple GRs. In most cases, GR was between 1 and 4 mm of severity (46.6%). The prevalence of GR ≥ 1 mm, ≥ 3 mm, and ≥ 5 mm increased through the age groups, whereas this age-dependent distribution was less pronounced when considering GR ≥ 7 mm.

GR frequencies documented in the literature are largely heterogeneous with percentages ranging from 25% of regular dental clinic attendants in Sweden [24] to 99.7% of adults over 35 years in Brazil [15] and 100% of subjects aged from 18 to 35 years in the UK [29]. In line with the present data, the prevalence of buccal GR was estimated to be 57.9% for persons over 30 years of age in the NHANES III US survey [10], and 68.7% among Colombian adults between 18 and 75 years of age [12]. Data from Italy reported prevalence ranging between 39 and 64% among undergraduate dental students [30–32].

Although these differences may be in part attributed to methodological issues (partial recording protocols, convenience samples), it is reasonable to infer that they may be also explained by different age ranges of the cohorts, periodontal profile, possible ethnic/genetic determinants, oral hygiene habits, and exposure to risk factors.

The current data showed that RT1 recession affected 41% of the study population, while RT2 and RT3 recessions were found to be less prevalent with a frequency of 25.8% and 36.7%, respectively. Noteworthy, this is the second study using the RT system introduced by Cairo et al. [17]. In a recent national population-based survey, Romandini et al. [16] reported lower estimates of RT1 (12.4%), but higher frequency for RT2 and RT3 recessions (88.8% and 55.0%) among adults of 30 years and older living in the USA. This different pattern of recession between the two surveys may be related to the characteristics of the participants. Our cohort was less large, younger in age, composed of only Caucasians with lower educational status than the US sampled population. Furthermore, it was representative of citizens living in an urban industrialized area from North-West Italy. Previous evidence supported a different distribution of GR by geographic area with people living in rural areas displaying significantly greater maximum recession scores [29].

The prevalence of mid-buccal GR demonstrated great differences according to the type of teeth. In line with findings from previously published reports [10, 12, 15, 33], the most affected teeth were mandibular incisors, mandibular premolars, and maxillary molars, while the least affected ones were the maxillary incisors. In contrast, Sarfati et al. [13] did not find any specific distribution pattern for GR according to tooth types and Serino et al. [24] referred to incisors and canines as the most affected teeth in young subjects, and to premolars in older ones.

Interestingly, RT1 recession occurred mostly at the buccal surface of maxillary and mandibular premolars and maxillary canines, while RT2 and RT3 recessions on maxillary molars and mandibular incisors. This is supported by data from epidemiological studies indicating that posterior sites are more prone to periodontal disease and that interdental sites at incisor and molar teeth experience more severe attachment loss in periodontitis patients [34]. However, Romandini et al. [16] observed that RT1 recession was more prevalent on maxillary and mandibular incisors, and that RT2 and RT3 recessions were evenly distributed among the dentition with a slight predilection for mandibular premolars and molars.

With respect to the symmetry along the dental midline, we detected a higher prevalence of gingival recessions on the right side and Addy et al. [35] on the left side, while Romandini et al. [16] and Sarfati et al. [13] reported a quite symmetric distribution. Although this study lacks information on toothbrushing techniques and hand preference, Tezel et al. [36] reported that right-handed subjects had more GRs in the premolar and canine region of the upper and lower right jaws.

Multivariable logistic regression analysis was carried out to identify risk indicators for RT1 recession and for RT2 and RT3 recessions, which were pooled together because both were associated with interdental attachment loss. Older age, lower percentage of plaque, and high education were found to be significant risk indicators of RT1 recession, while older age, severe periodontitis, low education, and poor oral hygiene were associated with RT2 and RT3 recessions. It has been previously demonstrated that age is one of the main contributors for the development of buccal recessions [10, 13, 14, 16, 33] and the present findings provide additional evidence. This could be reflective of the cumulative periodontal tissue loss and exposure to environmental risk factors across the lifespan, although it cannot be ruled out that the aging process leading to low-grade systemic inflammation and immune senescence could contribute to the development and progression of periodontal tissue damage [37, 38].

It is noteworthy that percentages of FMPS > 60% were significantly associated with higher odds of buccal RT2 or RT3 recessions, while percentages < 30% with higher odds of buccal RT1 recessions. These results support the speculation of Löe et al. [39], which refers to two possible types of GR associated with good and poor oral hygiene practices. Although clinical evidences indicate a positive correlation between buccal GRs, high standards of home oral hygiene and traumatic toothbrushing [30, 33, 40, 41] in the present study data on toothbrushing methods and brush hardness were not available.

In line with previous epidemiological studies [12, 15], there was an association between educational level and recessions. This finding may be explained by the fact that educated individuals are more aware of the importance of proper home plaque control and regular dental office attendance to maintain oral health conditions than their less-educated counterparts. Consistently, they are more prone to develop RT1 recessions, in contrast to moderately and lowly educated people who are more likely to experience RT2 or RT3 recessions.

Finally, considering that the RT classification system [17] refers to the loss of interproximal periodontal support, it is reasonable to consider RT2 and RT3 as a related phenomenon to advanced forms of periodontitis. Serino et al. [24] found that interdental attachment loss was associated with GR at the buccal surface and Yoneyama et al. [42] suggested that GR is the major feature of the progression of destructive periodontal disease with age. Indeed, this study suggests that the risk indicators for RT2 and RT3 recessions are similar to those traditionally associated to severe periodontitis [18]. We used the CDC/AAP periodontitis case definition, which was recommended in population-based epidemiological surveys [43]. Severe periodontitis was defined by the presence of both pathologic periodontal pockets and clinical attachment loss on interdental tooth surfaces [21, 22], allowing discrimination between periodontal atrophy and destructive periodontal disease [44].

The present study, although being the first survey using the RT classification in Europe, has some limitations. As this research was a secondary data analysis, the sample size was not calculated specifically to estimate GR prevalence. In addition, it was not possible to evaluate some variables inherent to oral hygiene methods and oral habits. Furthermore, neither the frequency of unidentifiable CEJ nor the different lesion morphologies of hard dental tissue in the cervical area were recorded [45]. In sites where CEJ was undetectable, we used the level of the CEJ on the adjacent teeth as reference [20]. Thus, we cannot rule out that the prevalence of shallow GRs (≤ 2 mm) could be underestimated. Lastly, this study is based on a representative sample of an industrialized city in North Italy; thus, findings cannot be generalized to other populations, and its cross-sectional design does not allow any conclusion regarding the temporality of the associations.

In conclusion, buccal GR is a fairly common finding in this Italian population and it is significantly associated to different contributing factors and tooth type distribution pattern according to the RT recession classes. This information can guide to identifying individual risk factor profiles and to implementing practical management and personalized prevention strategies for both clinical and surveillance purposes.

## Supplementary information

Appendix Fig. 1 Flow chart of the study (PNG 5394 kb)

High Resolution (TIFF 357 kb)

## Data Availability

The data that support the findings of this study are available from the corresponding author upon reasonable request.

## References

[1] Jepsen S, Caton JG, Albandar JM, Bissada NF, Bouchard P, Cortellini P, Demirel K, de Sanctis M, Ercoli C, Fan J, Geurs NC, Hughes FJ, Jin L, Kantarci A, Lalla E, Madianos PN, Matthews D, McGuire MK, Mills MP, Preshaw PM, Reynolds MA, Sculean A, Susin C, West NX, Yamazaki K (2018). Periodontal manifestations of systemic diseases and developmental and acquired conditions: consensus report of workgroup 3 of the 2017 World Workshop on the Classification of Periodontal and Peri-implant Diseases and Conditions. J Periodontol.

[2] Cortellini P, Bissada NF (2018). Mucogingival conditions in the natural dentition: narrative review, case definitions, and diagnostic considerations. J Clin Periodontol.

[3] Griffin SO, Griffin PM, Swann JL, Zlobin N (2004). Estimating rates of new root caries in older adults. J Dent Res.

[4] Wagner TP, Costa RS, Rios FS, Moura MS, Maltz M, Jardim JJ, Haas AN (2016). Gingival recession and oral health-related quality of life: a population-based cross-sectional study in Brazil. Community Dent Oral Epidemiol.

[5] Teixeira DNR, Zeola LF, Machado AC, Gomes RR, Souza PG, Mendes DC, Soares PV (2018). Relationship between noncarious cervical lesions, cervical dentin hypersensitivity, gingival recession, and associated risk factors: a cross-sectional study. J Dent.

[6] Chambrone L, Salinas Ortega MA, Sukekava F, Rotundo R, Kalemaj Z, Buti J, Pini Prato GP (2018) Root coverage procedures for treating localised and multiple recession-type defects. Cochrane Database Syst Rev 10(CD007161). 10.1002/14651858.CD007161.pub3 10.1002/14651858.CD007161.pub3 PMC6517255 30277568

[7] Stefanini M, Marzadori M, Aroca S, Felice P, Sangiorgi M (2000). Zucchelli G (2018) Decision making in root-coverage procedures for the esthetic outcome. Periodontol.

[8] Dai A, Huang JP, Ding PH, Chen LL (2019). Long-term stability of root coverage procedures for single gingival recessions: a systematic review and meta-analysis. J Clin Periodontol.

[9] Heasman PA, Holliday R, Bryant A, Preshaw PM (2015). Evidence for the occurrence of gingival recession and non-carious cervical lesions as a consequence of traumatic toothbrushing. J Clin Periodontol.

[10] Albandar JM, Kingman A (1999). Gingival recession, gingival bleeding, and dental calculus in adults 30 years of age and older in the United States, 1988–1994. J Periodontol.

[11] Chrysanthakopoulos NA (2013). Prevalence and associated factors of gingival recession in Greek adults. J Investig Clin Dent.

[12] Serrano C, Suarez E, Uzaheta A (2018). Prevalence and extent of gingival recession in a national sample of Colombian adults. J Int Acad Periodontol.

[13] Sarfati A, Bourgeois D, Katsahian S, Mora F, Bouchard P (2010). Risk assessment for buccal gingival recession defects in an adult population. J Periodontol.

[14] Holtfreter B, Schwahn C, Biffar R, Kocher T (2009). Epidemiology of periodontal diseases in the Study of Health in Pomerania. J Clin Periodontol.

[15] Rios FS, Costa RSA, Moura MS, Jardim JJ, Maltz M, Haas AN (2014). Estimates and multivariable risk assessment of gingival recession in the population of adults from Porto Alegre. Brazil. J Clin Periodontol.

[16] Romandini M, Soldini MC, Montero E, Sanz M (2020). Epidemiology of mid-buccal gingival recessions in NHANES according to the 2018 World Workshop Classification System. J Clin Periodontol.

[17] Cairo F, Nieri M, Cincinelli S, Mervelt J, Pagliaro U (2011). The interproximal clinical attachment level to classify gingival recessions and predict root coverage outcomes: an explorative and reliability study. J Clin Periodontol.

[18] Aimetti M, Perotto S, Castiglione A, Ferrarotti F, Mariani GM, Romano F (2015). Prevalence of periodontitis in an adult population from an urban area in North Italy: findings from a cross-sectional population-based epidemiological survey. J Clin Periodontol.

[19] Petersen PE, Ogawa H (2005). Strengthening the prevention of periodontal disease: the WHO approach. J Periodontol.

[20] Cairo F, Pini-Prato GP (2010). A technique to identify and reconstruct the cementoenamel junction level using combined periodontal and restorative treatment of gingival recession. A prospective clinical study. Int J Periodontics Restorative Dent.

[21] Page RC, Eke PI (2007). Case definitions for use in population-based surveillance of periodontitis. J Periodontol.

[22] Eke PI, Page RC, Wei L, Thornton-Evans G, Genco RJ (2012). Update of the case definitions for population-based surveillance of periodontitis. J Periodontol.

[23] Ravidà A, Troiano G, Qazi M, Saleh MHA, Saleh I, Borgnakke WS, Wang H-L (2020) Dose-dependent effect of smoking and smoking cessation on periodontitis-related tooth loss during 10 - 47 years periodontal maintenance - a retrospective study in compliant cohort. J Clin Periodontol 47:1132–1143. 10.1111/jcpe.13336 10.1111/jcpe.13336 32593185

[24] Serino G, Wennström JL, Lindhe J, Eneroth L (1994). The prevalence and distribution of gingival recession in subjects with a high standard of oral hygiene. J Clin Periodontol.

[25] Lang NP, Tonetti MS (2003). Periodontal risk assessment (PRA) for patients in supportive periodontal therapy (SPT). Oral Health Prev Dent.

[26] Trombelli L, Farina R, Silva CO, Tatakis DN (2018). Plaque-induced gingivitis: case definition and diagnostic considerations. J Clin Periodontol.

[27] Zucchelli G, Tavelli L, Ravidà A, Stefanini M, Suárez-López Del Amo F, Wang H-L (2018). Influence of tooth location on coronally advanced flap procedures for root coverage. J Periodontol.

[28] Zucchelli G, Tavelli L, Barootchi S, Stefanini M, Rasperini G, Valles C, Nart J, Wang H-L (2019). The influence of tooth location on the outcomes of multiple adjacent gingival recessions treated with coronally advanced flap: a multicentre re-analysis study. J Periodontol.

[29] Seong J, Bartlett D, Newcombe RG, Claydon NCA, Hellin N, West NX (2018). Prevalence of gingival recession and study of associated related factors in young UK adults. J Dent.

[30] Checchi L, Daprile G, Gatto MRA, Pelliccioni GA (1999). Gingival recession and toothbrushing in an Italian school of dentistry: a pilot study. J Clin Periodontol.

[31] Daprile G, Gatto MR, Checchi L (2007). The evolution of buccal gingival recessions in a student population: a 5-year follow-up. J Periodontol.

[32] Vignoletti F, Di Martino M, Clementini M, Di Domenico GL, de Sanctis M (2020). Prevalence and risk indicators of gingival recessions in an Italian school of dentistry and dental hygiene: a cross-sectional study. Clin Oral Invest.

[33] Susin C, Haas AN, Oppermann RV, Haugejorden O, Albandar JM (2004). Gingival recession: epidemiology and risk indicators in a representative urban Brazilian population. J Periodontol.

[34] Axelsson P, Lindhe J (1978). Effect of controlled oral hygiene procedures on caries and periodontal disease in adults. J Clin Periodontol.

[35] Addy M, Mostafa P, Newcombe R (1987). Dentine hypersensitivity: the distribution of recession, sensitivity and plaque. J Dent 15:242–248..

[36] Tezel A, Çanakçi V, Şiçek Y, Demir T (2001). Evaluation of gingival recession in left-and right-handed adults. Int J Neurosci.

[37] Baima G, Romandini M, Citterio F, Romano F, Aimetti M (2021) Periodontitis and accelerated biological aging: a geroscience approach. J Dent Res. 10.1177/00220345211037977 10.1177/00220345211037977 34609209

[38] Ebersole JL, Dawson DA, Emecen Huja P, Pandruvada S, Basu A, Nguyen L, Zhang Y, Gonzalez OA (2018). Age and periodontal health – immunological view. Curr Oral. Health Rep.

[39] Löe H, Ånerud Å, Boysen H (1992). The natural history of periodontal disease in man: prevalence, severity, and extent of gingival recession. J Periodontol.

[40] Toker H, Ozdemir H (2009). Gingival recession: epidemiology and risk indicators in a university dental hospital in Turkey. Int J Dent Hyg.

[41] Greggianin BF, Oliveira SC, Haas AN, Oppermann RV (2013). The incidence of gingival fissures associated with toothbrushing: crossover 28-day randomized trial. J Clin Periodontol.

[42] Yoneyama T, Okamoto H, Lindhe J, Socransky SS, Haffajee AD (1988). Probing depth, attachment loss and gingival recession. Findings from a clinical examination in Ushiku. Japan. J Clin Periodontol.

[43] Holtfreter B, Albandar JM, Dietrich T, Dye BA, Eaton KA, Eke PI, Papapanou PN, Kocher T, Joint EU/US Periodontal Epidemiology Working Group (2015). Standards of reporting chronic periodontitis prevalence and severity in epidemiological studies: proposed standards from the Joint EU/USA Periodontal Epidemiology Working Group. J Clin Periodontol.

[44] Hujoel PP, Cunha-Cruz J (2000). Selipsky H Saver BG (2005) Abnormal pocket depth and gingival recession as distinct phenotypes. Periodontol.

[45] Pini-Prato G, Franceschi D, Cairo F, Nieri M, Rotundo R (2010). Classification of dental surface defects in areas of gingival recession. J Periodontol.

