# Supplementary figures and images for: Estimates and multivariable risk assessment of mid-buccal gingival recessions in an Italian adult population according to the 2018 World Workshop Classification System
Source: Clin Oral Investig. 2022 Mar 18;26(7):4769–80. doi: 10.1007/s00784-022-04441-w (PMC9276566; doi:10.1007/s00784-022-04441-w)

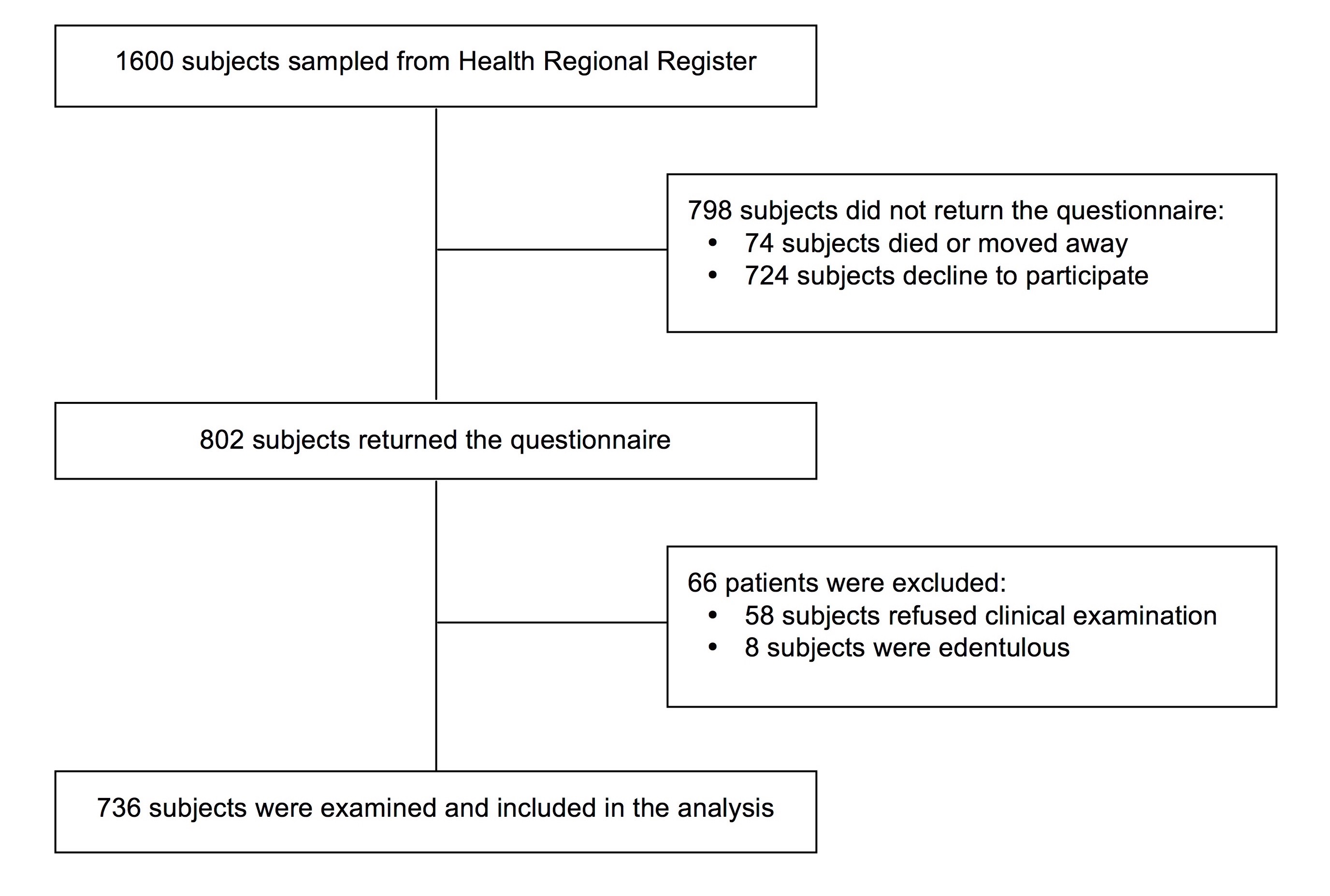

Supplement: Supplementary file 2 — High Resolution (TIFF 357 kb) [file 784_2022_4441_MOESM1_ESM.tiff]
